# Combined ultrasound and photoacoustic C-mode imaging system for skin lesion assessment

**DOI:** 10.1038/s41598-023-44919-5

**Published:** 2023-10-20

**Authors:** Anatoly Fedorov Kukk, Felix Scheling, Rüdiger Panzer, Steffen Emmert, Bernhard Roth

**Affiliations:** 1 Hannover Centre for Optical Technologies, Leibniz University of Hannover, Nienburger Straße 17, 30167 Hannover, Germany; 2 Clinic and Policlinic for Dermatology and Venereology, University Medical Center Rostock, Strempelstraße 13, 18057 Rostock, Germany; 3 Cluster of Excellence PhoenixD (Photonics, Optics and Engineering - Innovation Across Disciplines), Welfengarten 1a, 30167 Hannover, Germany

**Keywords:** Cancer imaging, Photoacoustics, Imaging and sensing, Biomedical engineering

## Abstract

Accurate assessment of the size and depth of infiltration is critical for effectively treating and removing skin cancer, especially melanoma. However, existing methods such as skin biopsy and histologic examination are invasive, time-consuming, and may not provide accurate depth results. We present a novel system for simultaneous and co-localized ultrasound and photoacoustic imaging, with the application for non-invasive skin lesion size and depth measurement. The developed system integrates an acoustical mirror that is placed on an ultrasound transducer, which can be translated within a flexible water tank. This allows for 3D (C-mode) imaging, which is useful for mapping the skin structure and determine the invasion size and depth of lesions including skin cancer. For efficient reconstruction of photoacoustic images, we applied the open-source MUST library. The acquisition time per 2D image is <1 s and the pulse energies are below the legal Maximum Permissible Exposure (MPE) on human skin. We present the depth and resolution capabilities of the setup on several self-designed agar phantoms and demonstrate in vivo imaging on human skin. The setup also features an unobstructed optical window from the top, allowing for simple integration with other optical modalities. The perspective towards clinical application is demonstrated.

## Introduction

The past several decades have been showing an increased incidence of skin cancer globally, which is likely caused by the increased leisure times of the population in zones with higher sun and UV exposure. Skin cancer and in particular melanoma, is problematic since it is visually often indistinguishable from normal birthmarks and moles, and in addition presents very little or no symptoms in the early stages. However, if left untreated, they can reach blood vessels and build metastases in the lymph nodes. As a result, the 10-year survival rate decreases from 95%, if discovered early, to less than 10% in the last stages with metastatic melanoma^1–4^.

In general, once skin cancer is clinically suspected the lesion has to be completely excised and histologically examined. If melanoma is diagnosed histologically, a second surgical procedure is required for re-excision with a 1 or 2 cm safety margin. For this procedure, the surgeon requires knowledge of the invasion depth and, thus, the vertical lesion size. To date, the exact lesion depth can only be measured histologically according to Breslow’s thickness metrics ^5^. At the moment, the gold standard for diagnosis, i.e. the first step in the procedure, consists of a complete lesion excision without a safety margin followed by histological examination of the lesion. This however is not only a painful and intimidating procedure for the patient, but also time and resource consuming. In addition, as it takes up to a few days for obtaining the corresponding diagnosis, it results in delay of the treatment, since several appointments and excisions are required in the cancer-positive case. If the dermatologist had the prior knowledge of the invasion depth and size, the tumor excision could be performed in one step at the first appointment.

As a result, numerous studies have tested different non-invasive imaging methods for measurement of skin lesions. For example, several studies have shown good agreement of high-frequency ultrasound (HFUS). Since there are discrepant definitions of HFUS, we apply the definition of Levy et al., who defines HFUS for frequencies $\ge$ 10 MHz^6^. with histological results for melanoma thicknesses^7–9^. At the same time, some of these studies point out the insufficient contrast of HFUS for melanoma depth measurement due to the small difference in the acoustical reflectivity between tumor and the surrounding skin tissues^10–12^.

Purely optical imaging methods, exploiting light scattering or absorption as contrast mechanisms, such as optical coherence tomography (OCT), confocal, fluorescence and multiphoton microscopy were also tested for imaging of skin lesions, yet found that their penetration depth is limited to $\le$1 mm due to the strong light scattering and absorption in highly pigmented skin^7,13,14^. The same is true for pure imaging concepts which usually have access to the upper skin layers only^15^.

Finally, photoacoustics (PA, sometimes referred to as optoacoustics, OA) has been emerging in dermatological imaging. Numerous publications in the last years demonstrate the high interest of researchers in the field of biomedical imaging, especially in the context of the photoacoustical microscopy ^16–21^. Compared to the conventional ultrasound imaging, where the image is based on the acoustical reflectivity of the sample, the contrast of PA is based on the optical absorption, which can be useful for determining the depth of melanoma^22–25^. PA can be classified into 2 sub-modalities: photoacoustical microscopy (PAM) and photoacoustical tomography (PAT).

PAM delivers *en face* images at a fixed depth, which is achieved by focusing both the pulsed excitation laser and the acoustical receiver to a co-localized spot at the sample and scanning in the lateral directions. This technique allows imaging at depths of several millimeters and features high spatial resolutions on a micrometer level^26,27^, which has also been applied for melanoma detection in vivo^25,28,29^. It is also a very popular technique for imaging of vascular structures due to the high contrast in optical absorption of blood. However, PAM acquires one pixel/voxel at a time, which usually results in extended measurement times, reaching as much as 20  min^30^ and consequently, requiring a very high number of laser pulses. The latter is problematic, since it can cause the measurement to exceed the legal Maximum Permissible Exposure (MPE) on human skin^31,32^. For example, Rule 3 of MPE regulations in Germany limit the energy density of every pulse to $20\text{mJ}/\text{cm}^2\cdot N^{-0.25}$, where *N* is the total number of pulses in a pulse train if the period between pulses is less than 18 µs. While this rule does not apply to skin in American ANSI regulations^31^, Rule 2 (total radiant exposure) still applies, which significantly restricts the application of high repetition pulses. However, this rule is rarely mentioned in publications. Thus, in many works on biomedical PAM the focus is on either artificial phantoms, ex vivo tissue or organs of small animals under anesthesia, but rarely for in vivo depth measurements on human skin.

On the other hand, PAT is done by either adding the axial scanning to PAM or by time-resolving the obtained signals from a multi-element transducer array ^33,34^. The reported works demonstrated PAT applied to animal tissue at depths up to 12 cm^35^. Since the transducer arrays are not optically transparent, the excitation pulses are delivered either coaxially using an acoustic-optical coupler^18,36^ or by illuminating from the side^24,37,38^. While PAT usually shows lower resolution than PAM, the acquisition is faster and requires less excitation pulses.

As an alternative solution for imaging of skin lesions, we present a novel 3D imaging system that integrates co-localized HFUS and PAT in a single measurement head. The proposed design involves a translatable acoustical mirror within a water tank with a 10 mm x 10 mm opening, allowing for optical excitation from the top. The measurement of a single depth plane is fast (<1 s) and with a pulse train of 4 pulses falls under the American and German MPE limits. The acoustical mirror is translatable within the tank, allowing for either precise positioning of the measurement plane or for C-mode imaging by using sequential steps with the attached motorized stage. Since the setup has an unobstructed optical view from the top, it can be easily integrated with other optical modalities, where Raman spectroscopy (RS) for melanoma dignity has the greatest potential^39–41^. The axial and lateral resolutions of each modality are measured on several self-designed phantoms and the setup is validated with preliminary measurements of in vivo imaging on human skin that are compared with histological measurement.

## Materials and methods

### Experimental setup

**Figure 1:**
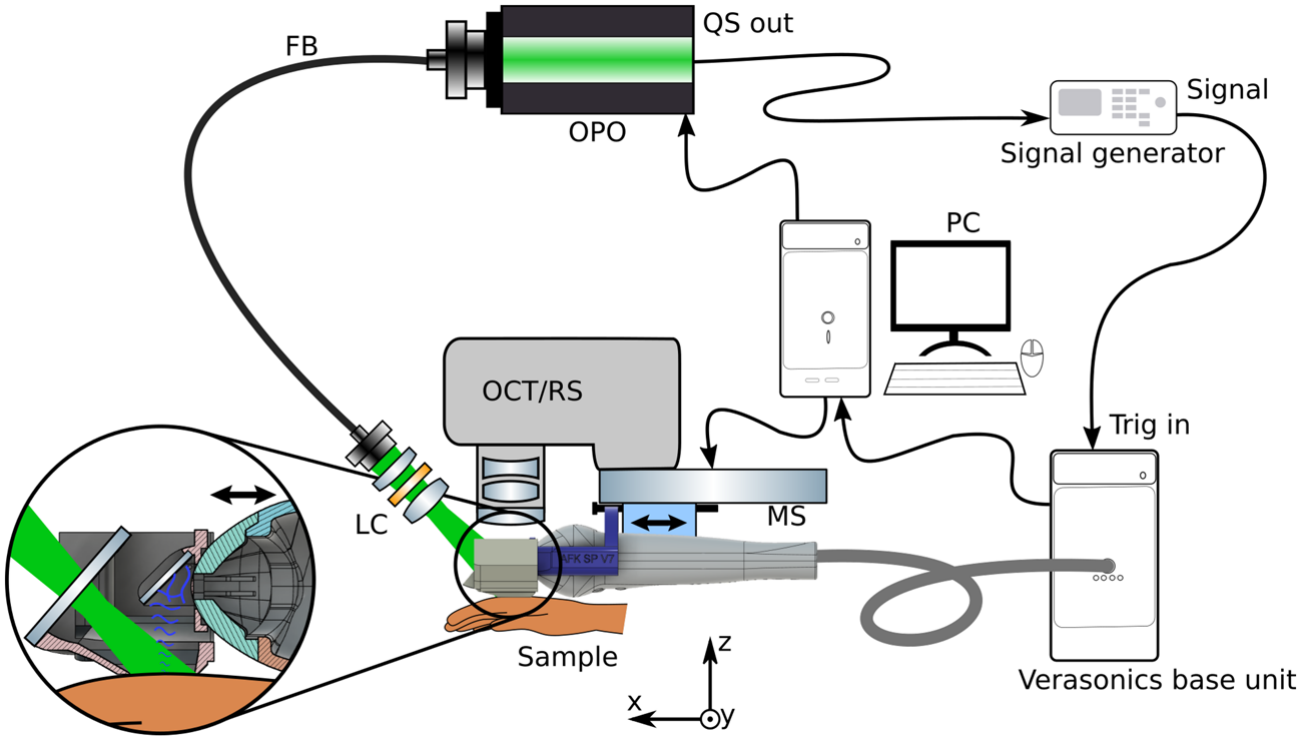
Sketch of the experimental setup. *FB* fiber bundle, *LC* light collimator, *MS* motorized stage. The US and PA perform B-mode imaging in the y-z plane; by translating the adapter in x direction with defined steps the C-mode is achieved. A photograph and a rendered sketch of the water tank with adapter are shown in Fig. 2.

**Figure 2:**
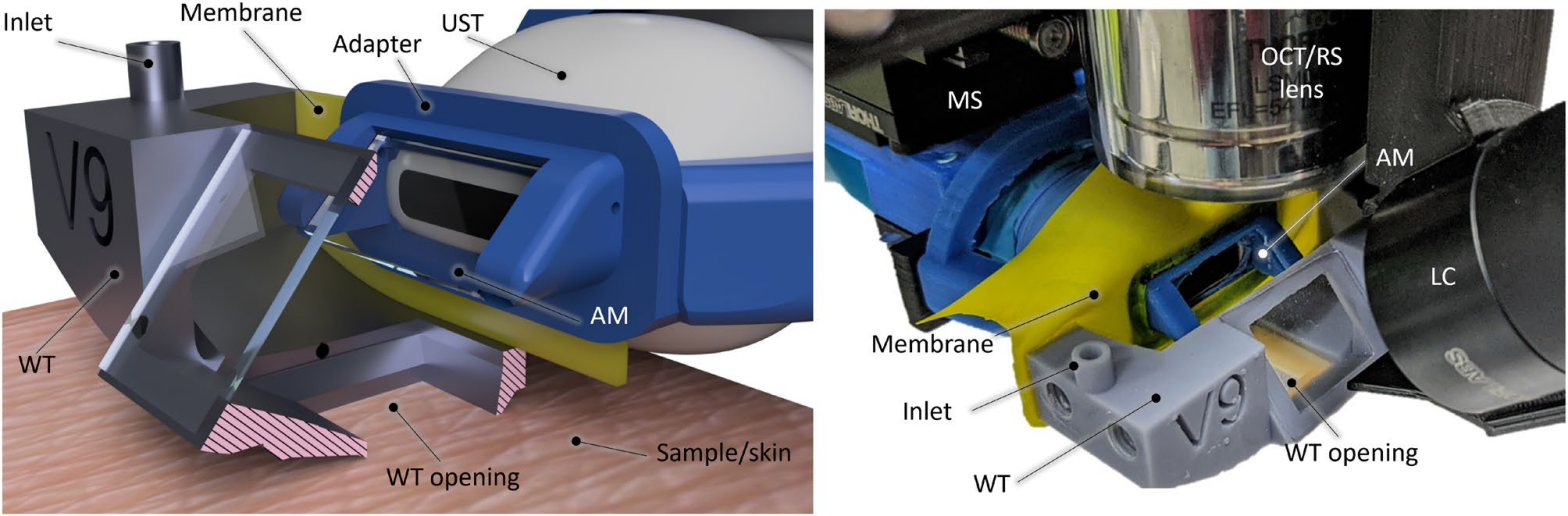
Left: rendered 3D image of the WT and UST adapter, being positioned on skin (without the OCT/RS setup). The WT is cut in the middle for a better view of the opening and the adapter, revealing the acoustical mirror (AM). Right: photograph of the US/PA setup being placed under the RS/OCT lens. The displayed inlet features a narrow water channel that runs through the WT and terminates just above the opening, making it easier to fill and drain the WT.

The schematic of the proposed design are depicted in Fig. 1. It is a modified part of the previously demonstrated multimodal setup for skin cancer detection and imaging, which additionally included OCT and Raman spectroscopy^42^. The reception and transmission of ultrasound is done with a single crystal transducer (L22-14vX-LF, Vermon, France). This transducer (UST) has 128 elements, 18 MHz central frequency with 67 % bandwidth (− 6 dB) and elevation pitch (acoustical focal length) of 20 mm. The transducer is connected to the research ultrasound system (Vantage 32LE, Verasonics, USA) via a Universal Transducer Adapter (UTA 260-MUX, Verasonics, USA), which is responsible for the data acquisition (DAQ) and ultrasound transmission, with 62.5 MHz sampling frequency. To ensure that OCT and US perform the measurements on the same position, a self-made water tank (WT) and UST adapter attachment are designed and printed on commercial 3D printers with acrylonitrile butadiene styrene (ABS) and resin, as shown in Fig. 2. The opening of the WT is positioned and pressed against the sample or skin area; the other part of the adapter is fixed on the UST. It carries a 1 mm thick microscope glass slide at 45$^\circ$ angle. When the WT is filled with water, the glass acts as an acoustical mirror (AM), reflecting acoustic waves by total internal reflection, which occurs due to the significant difference in the speed of sound between water and glass. The acoustical reflection efficiency was measured by comparing the US intensity with and without AM at the same distance. The negligible difference observed indicates a highly (> 95%) efficient reflection. The gap between WT and the adapter is sealed with a membrane made of 150 µm thick resistance band, allowing for waterproof motion of the UST in x direction and therefore scanning. The UST is attached to a motorized stage MS(MTS25/M-Z8, Thorlabs, USA) which allows for the translation or precise scanning of the B-mode imaging plane at the different positions of the WT opening. Moving the UST back enables optical access to the opening of WT from above and facilitates its combination with other optical modalities, such as OCT and RS. These modalities are currently being studied in other publications^41^.

The optical excitation is realized with an optical parametric oscillator (OPO, SpitLight Compact 400 OPO, InnoLas Laser GmbH, Germany), which emits pulses of 7 ns duration at 20 Hz repetition rate, with tunable wavelength within the VIS range. For simplicity of the present study, the wavelength is set to 430 nm, where the highest energy output is produced. The pulses are guided via a custom-made fiber bundle (CeramOptec GmbH, Germany), with numerical aperture of 0.22. The output surface of the fiber bundle ($\oslash ~=~6$ mm) is projected onto the opening of the WT with a light collimator (LC) consisting of 2 achromatic lenses (AC127-019-A and AC254-030-A, Thorlabs, USA) and a circle diffuser (ED1-C20-MD, Thorlabs, USA) which increases the beam intensity homogeneity at the WT opening. The laser illumination is performed at an angle of 50$^\circ$ from the x-y plane (sample surface) through the glass at the front of the WT. The synchronization for PA is done by connecting the Q-switch output of the OPO to the trigger input of a function generator (DS345, Stanford Research Systems, USA), which starts the ultrasound acquisition system with a negligible delay. The signal generator is implemented to reduce the excessively high trigger output voltage (10 V) from the OPO, and to reduce parasitic electronic signal caused by the flashlamp which resulted in notable noise on the US and PA images.

The measurement head, including the OCT/RS optics, is positioned on a commercially available articulated monitor arm for easy placement over skin lesions. After positioning, the WT is gently positioned on the skin, and the inlet for US/PA measurements is filled with water. The same inlet is later used to drain the tank. Because the WT fixes the lesion position at its opening, subsequent OCT or other imaging modalities can be utilized at the same location and later be colocalized.

**Figure 3:**
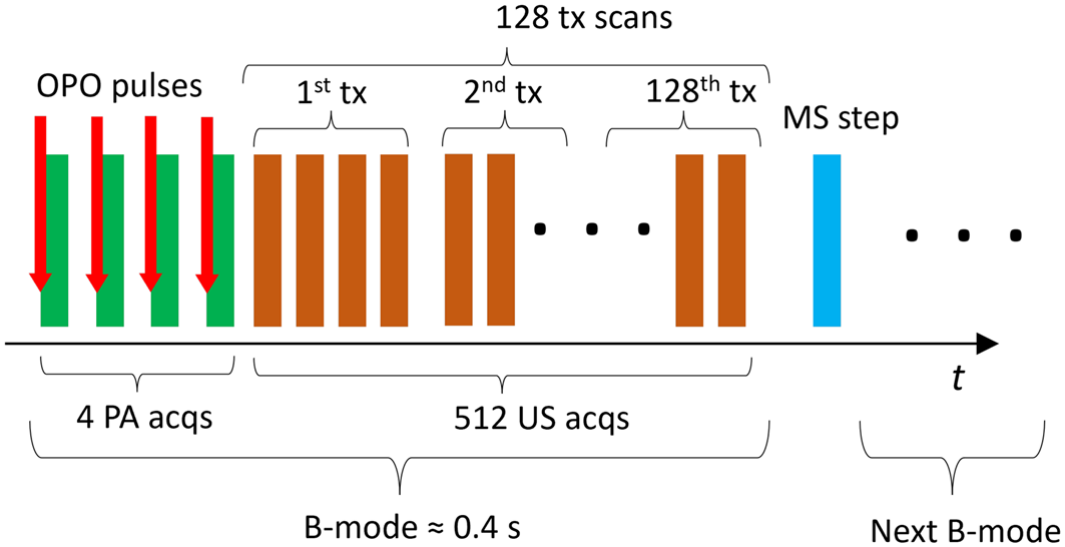
Sketch of the PA/US data acquisition sequence. First, the 4 PA measurements are performed, which are followed by 512 measurement events, which complete a full B-mode image. The MS motion and the OPO pulse release is done with a Python script.

The sequence of PA and US acquisition is depicted in Fig. 3. During a single B-mode measurement, the PA acquisition is performed first, followed by the US measurement at the same plane, which is achieved by transmitting a focused narrow beam to scan the sample surface 128 times in the y-direction. As the Vantage32 system used in this study can only simultaneously receive 32 channels, each measurement acquisition is performed four times to obtain the full aperture. In the current configuration, a single PA B-mode measurement is performed with <10 mJ/cm$^2$ energy density with 1 cm$^2$ WT opening per pulse. This is below the German and USA regulated MPE levels for human skin in vivo, which is calculated at 14 mJ/cm$^2$ per pulse for a sequence of 4 pulses^31,32^. Thus, a single combined PA/US B-mode measurement consists of 4$\cdot$(1+128)=516 consequent acquisition events. The movement of the stage and the laser firing for photoacoustic imaging are controlled by a Python script. A single B-mode measurement takes less than 0.4 seconds and is primarily constrained by the OPO repetition rate. With the inclusion of step translation with the MS for C-mode, the average acquisition speed is one slice per second.

### Resolution and agar phantoms

**Figure 4:**
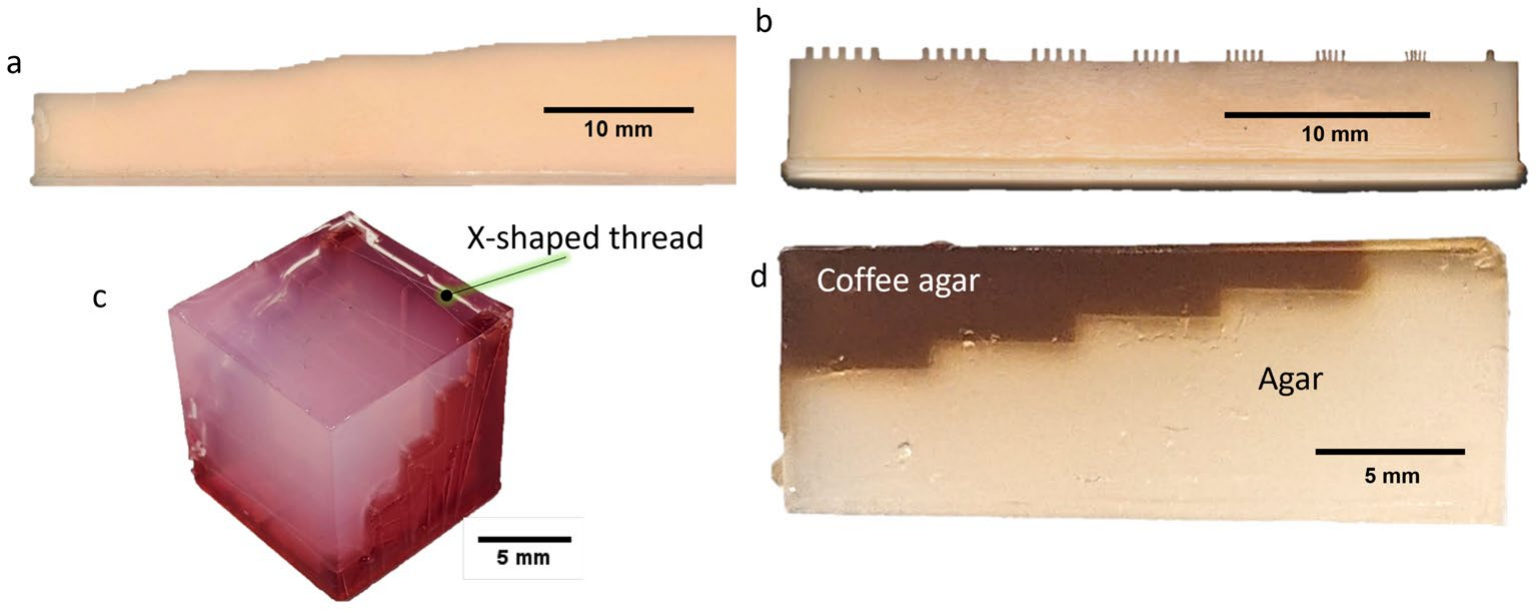
Image of agar phantoms for resolution measurements. (**a**) The “stairs” phantom with different step sizes; (**b**) the phantom with grooves for lateral resolution measurement; (**c**) cube agar phantom with floss strain in X shape at different depths; (**d**) the “coffee stairs” agar for demonstration of PA depth.

Since there is no clear consensus on how to measure the spatial resolution of the PA and US^43^, we have compared several approaches. For the presented study, the imaging capabilities were tested on 4 different self-made phantoms. For each phantom a specific mould is designed and printed on a commercial resin 3D printer, which allows geometrical accuracy of 50 µm. The first three moulds are then filled with deionized water. The fourth phantom, which is designed to demonstrate imaging penetration depths, is made of agar, which is a popular tissue mimicking substance for ultrasound and photoacoustic imaging^44–46^. The agar mixture is prepared by heating 100 ml of deionized water to boiling temperature inside an Erlenmeyer flask with constant magnetic stirring and adding 2 g of agar powder (A1296, Merck KGaA, Germany). The solution was magnetically stirred at 80$^\circ$ temperature for about 5 min and injected into the mould. To create a smooth upper surface, the top part of the mould is covered with a microscope slide. After cooling for 2 h at room temperature, the phantom is solidified enough for measurements. The different phantoms are shown in Fig. 4.

The first phantom (Fig. 4a is designed to measure the axial resolution with “stairs” of different step heights. For measurements of the lateral resolution, a second phantom (Fig. 4b with several gratings with periods between 100 µm and 800 µm is used, similar to a USAF resolution target. For both phantoms, the resolution is considered as the smallest distinguishable grating step/period size. The third phantom(Fig. 4c is a cube with notches on the diagonal side, where a single thread from dental floss is positioned in a crossed shape at different depths. By performing C-mode imaging with steps of 100 µm, the resolution is measured with Sparrow’s resolution criterion, which is defined by finding the smallest separation between two threads at which they start to become distinguishable (identified by the emergence of the dip between the peaks). In addition, the separate threads were also used to image as point spread functions to measure the full width at half maximum (FWHM) as an additional resolution criterion, since the measured thickness of the thread ($\approx$ 20 µm) is significantly smaller than the general spatial resolution of the system. The last phantom (Fig. 4d is intended to demonstrate the depth measurement capabilities of PA modality. This phantom consists of 2 agar stairs structures, which are fitted into each other. The upper structure imitates a skin mole with different depths, and it consists of agar mixed with coffee. The latter acts as the absorbing agent, since it is known that coffee has similar optical absorption properties as melanin^47,48^. The coffee agar is prepared by replacing the distilled water in the agar receipt with coffee solution, which is prepared by cooking 1 l of water with 5 full tablespoons of standard coffee powder inside a French press.

### Reconstruction of PA

Although the Verasonics system has a fast and efficient image reconstruction system, it is less suitable for the reconstruction of PA images. For example, this method does not allow compensation for time delays caused during Q-switch triggering, or adjustment of the f-number of the receiving aperture. These limitations can negatively impact resolutions and may prove difficult to implement in multiwavelength acquisitions in the future. The problem was addressed by conducting a PA reconstruction using the open-source MATLAB library MUST^49,50^. The reconstruction approach involves demodulating the raw PA signals into in-phase and quadrature components (IQ) and multiplying them with a sparse DAS matrix to generate the images (although it is not mentioned in the documentation of MUST library, it allows for one-way reconstruction by setting param.passive = True.). The receiving f-number for PA is set to 0.5 using the parameter grid search, which results in the best visible resolution on the grooves and stairs phantom. In order to match the US images, the PA was reconstructed with 25 µm sized pixels. Following the reconstruction, an edge detection algorithm was employed to find the time delay between US and PAT acquisitions, which enables overlaying the images, as shown in Fig. 8.

### In vivo samples

The setup was validated in vivo on three skin nevi of three human volunteers. The performed measurements were approved by the Ethics Committee of the University Medical Center Rostock (A 2016-0115) and met the principles of the Declaration of Helsinki. Informed consent was obtained from all participants in this study, in oral and written form. The WT was positioned roughly at the center spot of the nevus and the C-mode PA/US was performed with 20 slices with separation of 200 µm. Immediately afterwards the nevi were excised and embedded in 10 % formalin solution for histological examination of hematoxylin-eosin (H&E) stained slices. The maximal nevi depth (comparable to Breslow’s depth in melanoma) was identified and measured by a dermato-pathologist.

## Results and discussion

**Figure 5:**
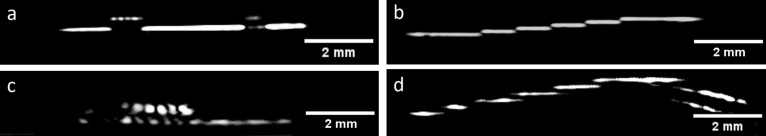
The results for resolution measurement on grooves and stairs phantoms. The smallest period/step sizes that could still be distinguished are presented. (**a**) Smallest groove period (200 µm, at the left side) and (**b**) step size of 100 µm, measured with US; (**c**) period of 300 µm  and (**d**) step of 200 µm, measured with PA, respectively.

**Figure 6:**
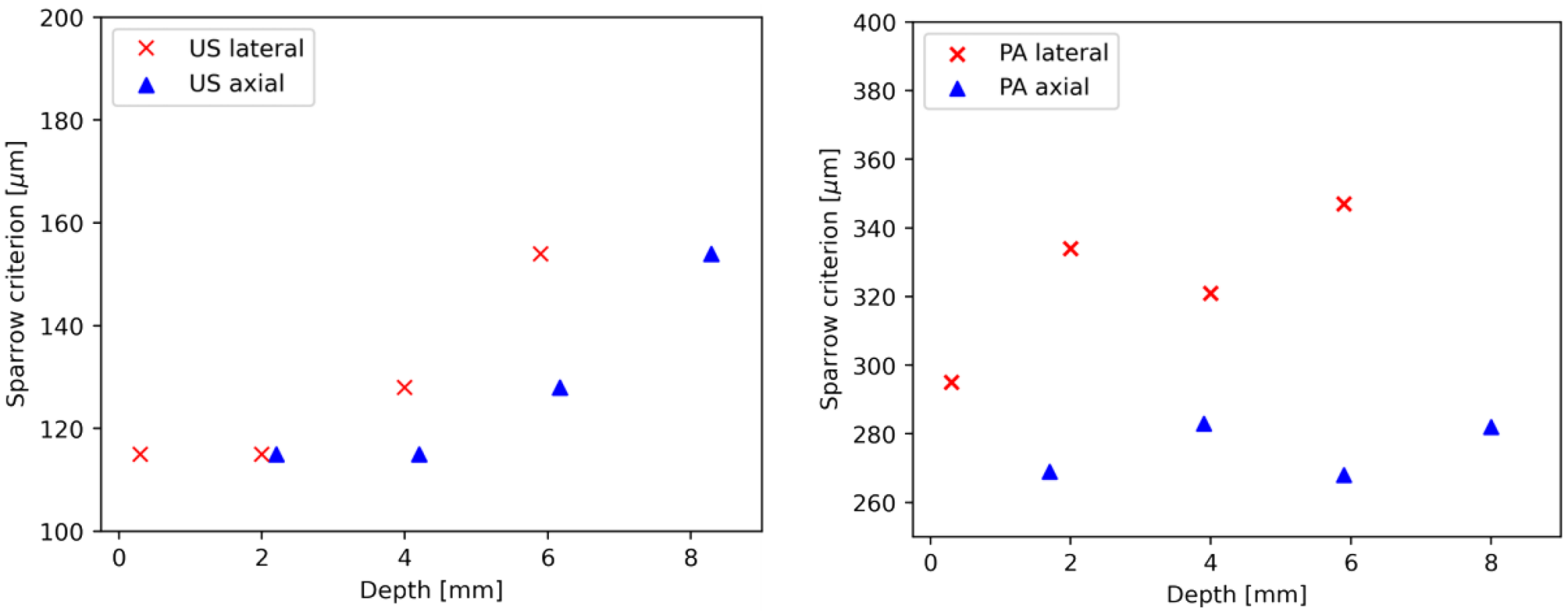
Results for the experimentally determined resolution according to Sparrow’s criterion (the smallest separation at which 2 strains can be distinguished) at different depths inside the cube phantom. Left: results for US, right: results for PA. Note: the starting depth of 0 mm is the position at the acoustical focal plane of the transducer, i.e. at 20 mm.

In a first set of measurements, the performance of the combined system was characterized with respect to resolution by using the designed agar phantoms. The results for the resolution measurement with grooves and stairs phantoms are presented in Fig. 5. For US, the lateral and axial resolutions are measured at 200 µm and 100 µm respectively; for PA this resulted in 300 µm and 200 µm, respectively. The resolution measurements according to Sparrow’s criterion (phantom with X-crossed strains, see Fig. 4c) predict similar values, which are presented in Fig. 6. This shows that co-localized measurements on samples can be performed with a resolution that is useful for the dermatological examination on skin.

**Figure 7:**
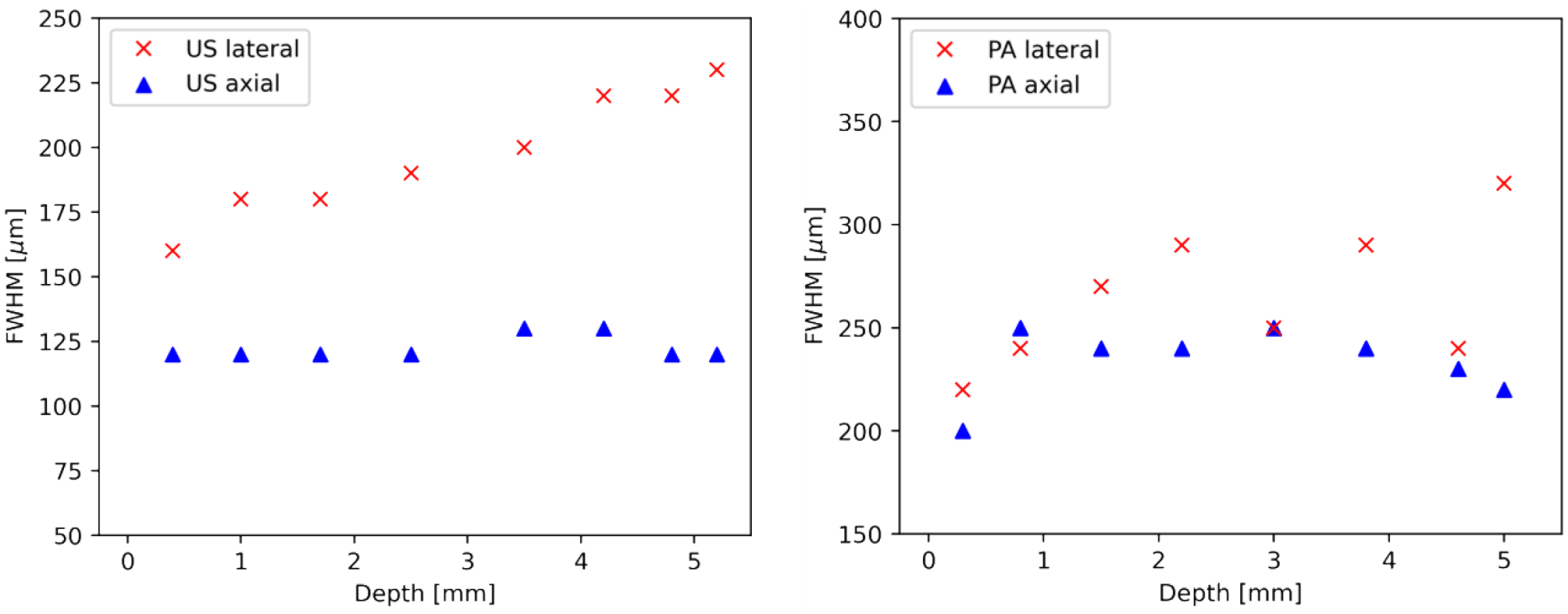
Results for FWHM of US on a single 20 µm thick strain measured at different depths inside an agar phantom. Left: results for US, right: results for PA. Note: the starting depth of 0 mm is the position at the acoustical focal plane of the transducer, i.e. at 20 mm.

Furthermore, the FWHM of single strains incorporated in the agar phantoms at different depths were measured, similar to common wire phantom. The results obtained are presented in Fig. 7. The FWHM is measured automatically by fitting the plot profile of the uncompressed intensity (modulus of the beamformed IQ) pixels with a Gaussian function. On average, the axial resolutions for both PA and US remain constant along the axial direction, while the lateral resolutions decrease. This trend and the average results are consistent with the results of similar reported US/PA systems^51^.

Overall, the resolution results show agreement with each other, predicting an average US resolution of < 200 µm and PA of < 350 µm. The latter is expectedly lower, since it can be viewed as single plane wave US imaging, whereas the US is a compound imaging approach realized with multiple acquisitions and allowing for reconstruction of finer structures.

**Figure 8:**
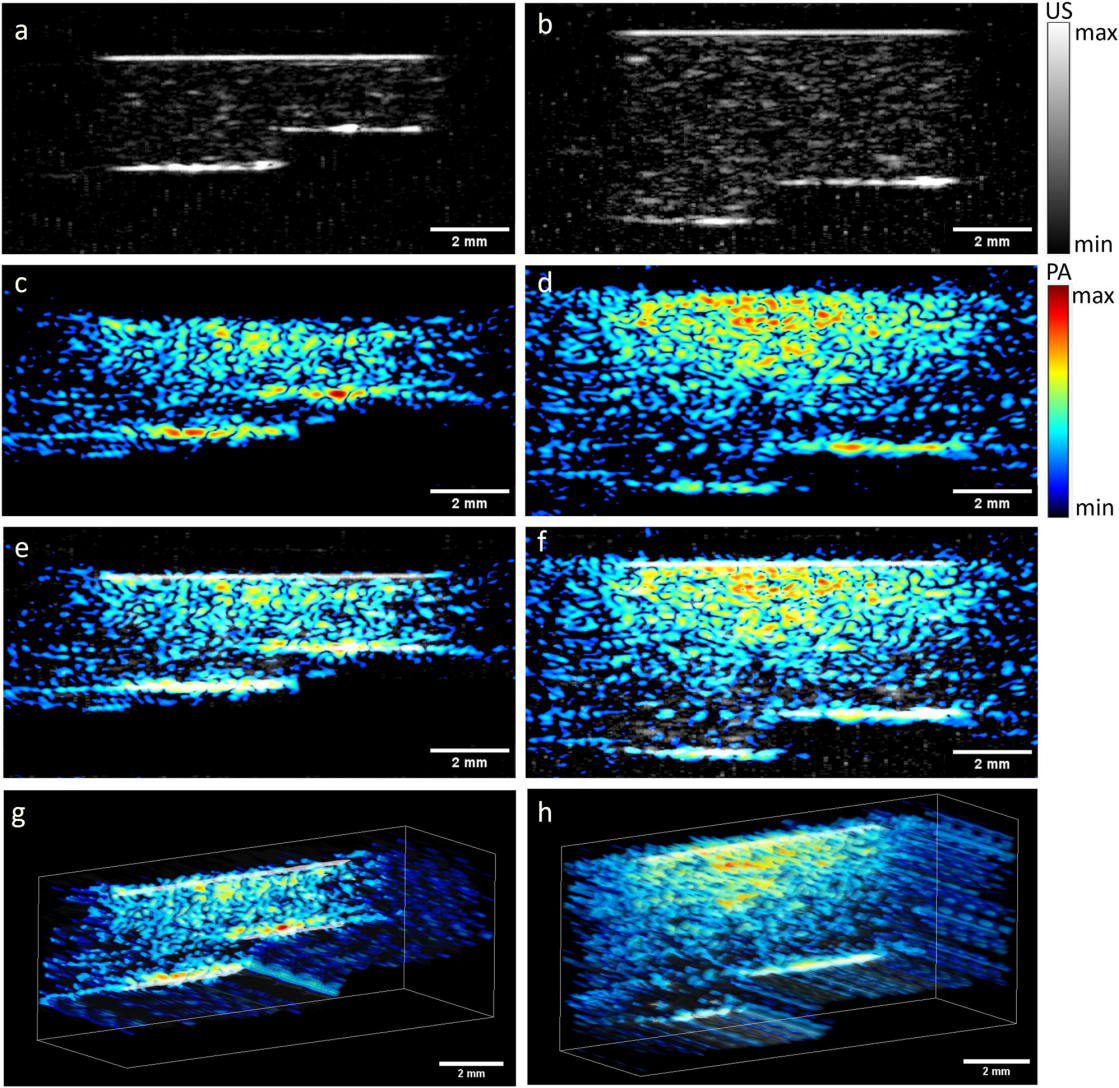
US and PA imaging with the coffee agar phantom. Left images are measurements of the 2–3 mm depth steps, right images: 4–5 mm depth. (**a**,**b**) US; (**c**,**d**) PA; (**e**,**f**) combined image with US and PA are shown in grayscale and false color, respectively; (**g**,**h**) C-mode representation of the phantoms. The edges of the images are blurred due to the shadowing effect from the 10 mm WT opening, which is more visible with the US imaging. The speckles on the US images are due to the electronic noise caused by the OPO functioning.

The imaging of the coffee agar phantom is presented in Fig. 8. The US shows stronger signal on the upper coffee part, which can be attributed to the remaining unfiltered coffee particles in the agar. The measurement demonstrates that PAT is capable of imaging structures as deep as 5 mm. Assuming that the absorption and scattering of pigmented skin nevi is similar, the system can be expected to resolve invasion depths of most skin lesions, including stage IV Breslow’s depth.

**Figure 9:**
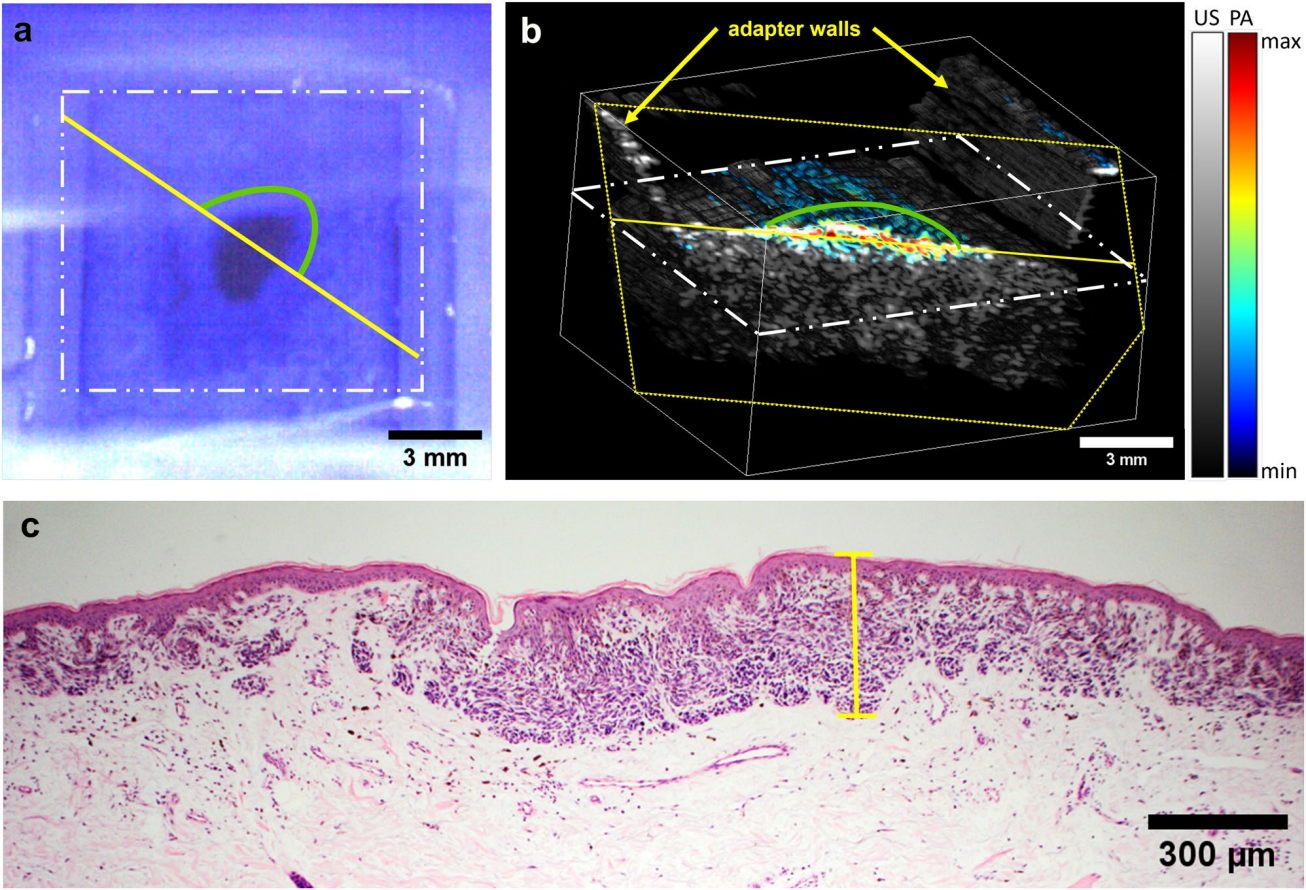
Example in vivo measurement on a human nevus. (**a**) The image of the skin lesion (dark spot at the center) placed inside the WT, taken from the OCT module before the US/PAT measurement. The white, yellow and the green lines mark the imaging area, clipping line and the approximate lesion position in (**b**) respectively. The dominance of blue color is caused by an optical filter of the OCT/RS module. The blurry horizontal line in the middle is the edge of the acoustical mirror positioned above the opening. (**b**) C-mode representation of the combined US/PA measurement, which is cut diagonally at the center of the lesion (the dashed yellow lines represent the cutting plane, which intersects the sample at the solid yellow line), revealing the PAT signal. US is shown in grayscale and PAT in false color. (**c**) The corresponding histological measurement, with yellow line indicating the position of the deepest melanocytic invasion (corresponding Breslow thickness) of 340 µm, measured by a histologist.

**Figure 10:**
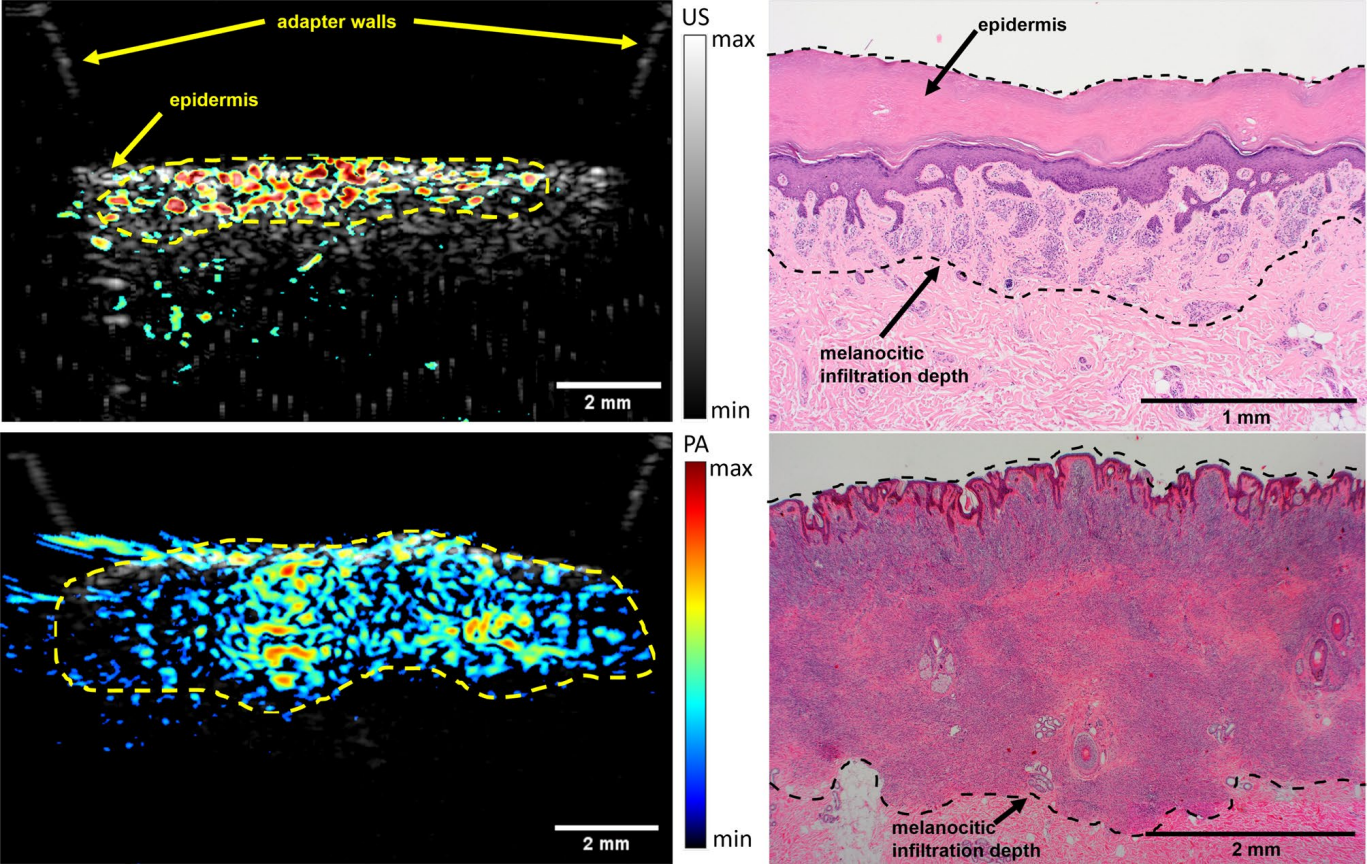
Comparison of combined in vivo US/PA measurement on human nevi (left) and the corresponding histological measurements (right). US is represented in grayscale and the PA in false color. The dash lines represent the manual measurement of the lesion borders. Top: a thin lesion from heel area with a Breslow thickness of 1 mm. Bottom: a thicker nevus from abdomen area with average 3 mm thickness and maximal depth of 3.5 mm.

The in vivo US/PAT measurements and the corresponding histological results on human volunteers are presented in Figs. 9 and 10. All three excisions were diagnosed by the dermatopathologist as melanocytic compound nevi. As seen in Fig. 9, the C-mode imaging allows to visualize the size and localize the position of the largest depth. The lesion is measured at 340 µm by the histologist, the corresponding value obtained from PA is slightly larger in this case (500 µm). Note that the difference might arise from the fact that histology may underestimate the thickness of the lesion due to tissue shrinkage and, in addition, it might not be taken at the same position. The second nevus, shown in Fig. 10, is from the heel area with a thick ($\approx$ 400 µm) stratum corneum (SC), which is also visible with the US. Similar to other findings, the US presents a much poorer contrast as compared to PAT when it comes to reveal the melanocytic invasion depth. On the other hand, the PA thickness (area of PAT signal above threshold value of 15% intensity at 20 dB), manually measured at several positions results in 984 ± 100 µm, correlating well with the histological invasion depth (including the SC) of 1009 ± 75 µm. The measurements of the third skin nevus from abdomen region also shows good agreement between PA at 2917 ± 310 µm and average histological thickness of 3023 ± 231 µm.

With the in vivo validation of the presented system, we believe that it is well capable of assisting the dermatologists in measuring the size of the lesion and deciding on the excision margins. The FWHM resolutions of our system are equivalent to or better than those of other US/PA devices reported in the literature, which are mostly based on commercial US systems and have been used in various applications, however, not for skin cancer so far^52–54^. In addition, the presented design features an optical window positioned above the measurement aperture, enabling seamless integration with other optical modalities without requiring switching of measurement heads. Our future studies aim to integrate the presented setup with a 900 nm Fourier domain OCT and a Raman spectrometer, which will share the optical excitation with the PAT. These additional two modalities will deliver further required information on the skin lesion, in particular on the pathophysiological properties (i.e. the dignity) which can be established from the morphological structure indicated with high resolution OCT and with the ratio of several characteristic Raman peaks, which is an indicator for malignant melanoma^55–58^. In addition, it is possible to take advantage of the wavelength tunability of the OPO and perform PAT with excitation at different wavelengths, enhancing the information of PAT to PA spectrometry^59^. The excitation at different wavelengths would also amplify the RS modality, by allowing to select the wavelength at which the sample fluorescence is minimized, and Raman peaks are better seen^60^. Finally, it is intended to test the more complex reconstruction techniques for PAT, such as Delay-Multiply-and-Sum (DMAS), double stage DMAS or minimum variance algorithms, which have been known to improve the image resolution and reduce the sidelobes, which are particularly present with DAS.

## Conclusion

We have developed a single-head co-localized US/PAT imaging system allowing for structural and depth measurement with the application for non-invasive diagnosis of skin cancer. Compared to the other reported systems, our design has also an unobstructed window for other optical modalities, short acquisition of <1 s per B-mode, C-mode capability and the energy pulse density is in accordance with MPE levels. The optical excitation is done with a wavelength-tunable OPO, allowing for multiwavelength PAT acquisition. The resolution of the system is measured at 200 µm lateral, 100 µm axial for HFUS and 300 µm lateral, 200 µm axial for PAT, respectively. The PAT measurement depth, determined on a skin phantom resembling skin lesions, is at least 5 mm. Finally, the setup is tested in vivo on a human volunteers, where C-mode measured dermal structure and melanocytic depth correlated well with the histology. Future work will focus on multiwavelength PAT analysis, improved PAT imaging with more complex beamforming algorithms and integration with other optical modalities such as OCT and RS for additional information on the dignity of skin lesions.

## Data Availability

The datasets generated during and/or analysed during the current study are available from the corresponding author on reasonable request.
